# Biocontrol characteristics of the fruit fly pupal parasitoid *Trichopria drosophilae* (Hymenoptera: Diapriidae) emerging from different hosts

**DOI:** 10.1038/s41598-018-31718-6

**Published:** 2018-09-06

**Authors:** Jiani Chen, Sicong Zhou, Ying Wang, Min Shi, Xuexin Chen, Jianhua Huang

**Affiliations:** 1 Institute of Insect Sciences, Zhejiang University, 866 Yuhangtang Road, 310058 Hangzhou, China; 2 Ministry of Agriculture Key Lab of Molecular Biology of Crop Pathogens and Insect Pests, Zhejiang University, 866 Yuhangtang Road, 310058 Hangzhou, China; 3 State Key Lab of Rice Biology, Zhejiang University, 866 Yuhangtang Road, 310058 Hangzhou, China

## Abstract

*Trichopria drosophilae* (Hymenoptera: Diapriidae) is an important pupal endoparasitoid of *Drosophila melanogaster* Meigen (Diptera: Drosophilidae) and some other fruit fly species, such as *D. suzukii*, a very important invasive and economic pest. Studies of *T. drosophilae* suggest that this could be a good biological control agent for fruit fly pests. In this research, we compared the parasitic characteristics of *T. drosophilae* reared in *D. melanogaster* (TD_m_) with those reared in *D. hydei* (TD_h_). TD_h_ had a larger size than TD_m_. The number of maximum mature eggs of a female TD_h_ was 133.6 ± 6.9, compared with the significantly lower value of 104.8 ± 11.4 for TD_m_. Mated TD_h_ female wasp continuously produced female offspring up to 6 days after mating, compared with only 3 days for TD_m_. In addition, the offspring female ratio of TD_h_, i.e., 82.32%, was significantly higher than that of TD_m_, i.e., 61.37%. Under starvation treatment, TD_h_ survived longer than TD_m_. TD_h_ also survived longer than TD^m^ at high temperatures, such as 37 °C, although they both survived well at low temperatures, such as 18 °C and 4 °C. Old-age TD_h_ females maintained a high parasitism rate and offspring female ratio, while they were declined in old-age TD_m_. Overall, TD_h_ had an advantage in terms of body size, fecundity, stress resistance ability and the parasitism rate compared with TD_m_. Therefore, *T. drosophilae* from *D. hydei* could improve biocontrol efficacy with enormous economic benefits in the field, especially in the control of many frugivorous Drosophilidae species worldwide.

## Introduction

*Drosophila* is a genus of flies belonging to the family Drosophilidae. Some species of *Drosophila* (also called fruit flies), particularly *D. melanogaster*, have been widely used in the research of genetics, developmental biology and human diseases^1,2^. However, some *Drosophila* species are destructive pests of agriculture, especially damaging soft fruits such as berries, cherries and wine grapes^3^. Fruit flies generally lay eggs in decaying fruits, and the larvae feed and develop with the fruits, which causes health risks and economic losses. Traditional chemical control methods for fruit flies have low efficiency and are harmful to public health^4^. Consequently, biological control with parasitoids is more sustainable and is urgently needed. Parasitic wasps constitute a major class of natural enemies of many agriculture pests and have tremendous value as biocontrol agents. Most known parasitoid wasp species attack the egg, larval or pupal stages of their hosts and they carry virulence and some other parasitic factors to modify hosts’ physiology and immunity, to change hosts’ metabolism, to destruct hosts’ endocrine and reproductive structures, and finally kill the hosts for their own development^5–8^. Many parasitoids are reported to attack various Drosophilidae species, and the majority of them are larval parasitoids, such as *Leptopilina heterotoma*, *L. boulardi* and *Asobara tabida*^9,10^. Recently*, Trichopria drosophilae* (Hymenoptera: Diapriidae), an important pupal endoparasitoid of *D. melanogaster* and some other fruit fly species, has been found to be an ideal natural enemy to constrain the fruit fly population because it has extremely high parasitism efficiency^11–14^. The life history and biological characteristics of *T. drosophilae* have been well studied by several groups. In 2012, Chabert *et al*. found that *T. drosophilae* was effective against many fruit fly species, including *D. suzukii*, a well-known invasive pest^12^. Female *T. drosophilae* emerged with a relatively high number of mature eggs, and the egg numbers increased during their first four days after eclosion. This indicates that *T. drosophilae* might maximize reproduction during early adult life^14^. Moreover, the parasitism rate of *T. drosophilae* is higher than that of another well-known cosmopolitan pupal parasitoid, *Pachycrepoideus vindemmiae* (Diptera: Pteromalidae)^13^. Although *T. drosophilae* is reported to be effective against *Drosophila* species under laboratory conditions, it is necessary to find the parasitoids that have the highest parasitism rate, highest female offspring numbers and longest adult longevity and which are resistant to certain stress conditions, such as food deprivation and extreme weather conditions, for the biological control purpose of augmentative release in the field.

To increase the effectiveness of parasitoids as natural enemies, female adult wasps are supplied with extra nutrient sources, such as sugars, to enhance their longevity and fecundity and subsequently, the biocontrol efficacy^15,16^. However, host quality can also have a major influence on the fitness and parasitic efficiency of offspring^17^. Lampson *et al*. found that different sizes of the same parasitoid had an effect on several biological characteristics, suggesting that larger parasitoids have a longer life span and greater competitiveness^18^. Another comparative study on the parasitism of *P. vindemmiae* hatching from housefly and fruit fly pupae showed a positive correlation between the size of the host and the size of the emerged offspring, as well as the longevity, the oviposition duration and other parasitic attributes^19^.

Based on the results of previous studies^14^, *T. drosophilae* reared on a larger sized host could be more advantageous for further biological control. Here, we used *D. hydei* as a substitute host, of which the pupae are significantly larger than those of *D. melanogaster*. Then, we compared the body size, fecundity, stress resistance ability and parasitism efficiency between the two parasitoid populations that emerged from the different hosts.

## Results

### The parasitoid and host size measurements

**Figure 1:**
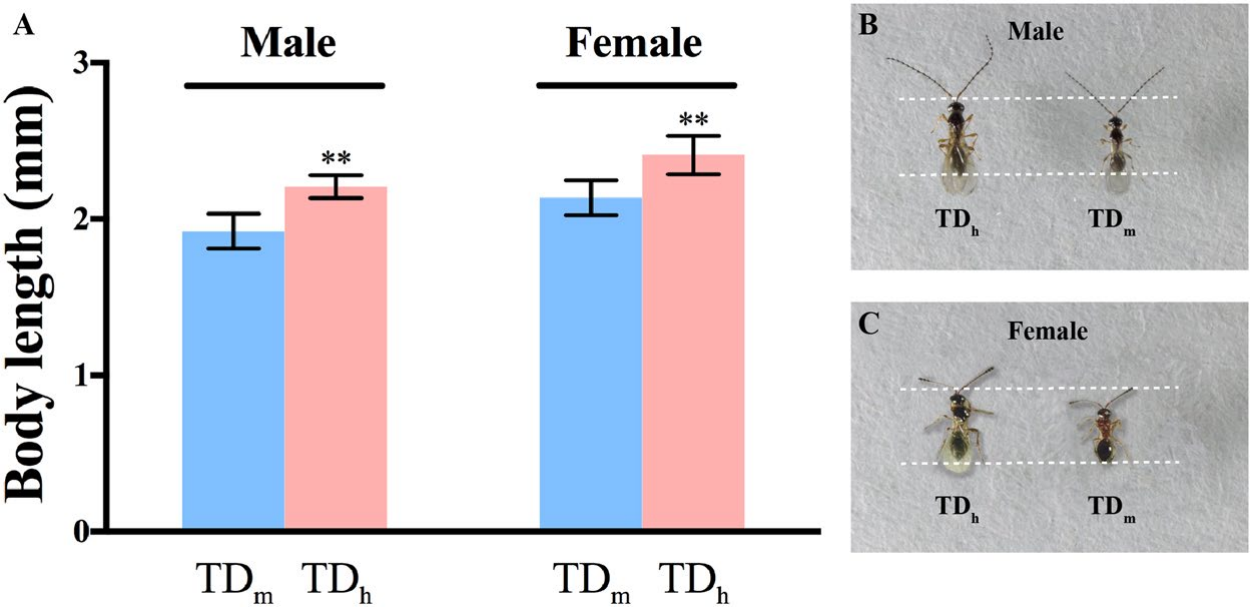
(**A**) Body length measurements for TD_h_ and TD_m_. The body length of female TD_h_ was 2.41 ± 0.12 mm (n = 10), compared with 2.12 ± 0.11 mm (n = 12) for female TD_m_ (t = 5.50, df = 20, P < 0.01). The body length of male TD_h_ was 2.21 ± 0.07 mm (n = 16), compared with 1.92 ± 0.11 mm (n = 10) for male TD_m_ (t = 7.94, df = 24, P < 0.01). Significant differences based on Student’s t-test at P < 0.05 are indicated by asterisks. (**B**) Photographs of male TD_h_ and TD_m._ The body length of parasitoids was measured as the length from the head to the tip of the abdomen. (**C**) Photographs of female TD_h_ and TD_m_.

The respective pupal length and width were 4.05 ± 0.13 mm and 1.27 ± 0.04 mm for *D. hydei* (n = 18) and 2.93 ± 0.14 mm and 0.99 ± 0.06 mm for *D. melanogaster* (n = 37). The size of *D. hydei* was significantly larger than that of *D. melanogaster* (length: t = 28.57, df = 53, P < 0.01, width: t = 18.68, df = 53, P < 0.01). To investigate whether there was a correlation between the size of the hosts and their offspring, *T. drosophilae* was used to parasitize *D. melanogaster* and *D. hydei* pupae. The measurements indicated that the body length of TD_h_ was significantly longer than that of TD_m_, in both females and males (Fig. 1A–C). The body length of female TD_h_ was 2.41 ± 0.12 mm (n = 10), compared with 2.12 ± 0.11 mm (n = 12) for female TD_m_ (t = 5.50, df = 20, P < 0.01). The length of male TD_h_ was 2.21 ± 0.07 mm (n = 16), compared with 1.92 ± 0.11 mm (n = 10) for male TD_m_ (t = 7.94, df = 24, P < 0.01). These results showed that the size of TD_h_ was much larger than that of TD_m_.

### Parasitism rate and offspring female ratio comparison

The results showed that this local 4-day old *T. drosophilae* females had an extremely high parasitism rate. Approximately 97% of the *D. melanogaster* pupae were successfully parasitized by TD_m_, and no significant difference in the parasitism rate was found between TD_h_ and TD_m_ (t = 1.67, df = 4, P > 0.05) females. However, the offspring female ratio of TD_h_, which averaged 82.32%, was significantly higher than that of TD_m_, which averaged 61.37% (t = 8.96, df = 4, P < 0.01) (Table 1).

**Table 1:** Parasitism rate and offspring female ratio of 4-day old female parasitoids that emerged from two different hosts.

Parasitoid	Number of hosts	Number of emerged flies	Parasitism rate	Average parasitism rate	Number of emerged wasps	Number of emerged female wasps	Offspring female ratio	Average offspring female ratio
TD_m_	200	1	99.50%	97.06 ± 1.86%	171	102	59.65%	61.37 ± 2.32%
	120	6	95.00%		99	64	64.65%	
	120	4	96.67%		102	61	59.80%	
TD_h_	200	0	100.00%	99.44 ± 0.79%	193	164	84.97%	82.32 ± 2.35%
	120	0	100.00%		106	84	79.25%	
	120	2	98.33%		110	91	82.73%	

TD_h_ and TD_m_ represent *T. drosophilae* that emerged from *D. hydei* and *D. melanogaster*, respectively. There was no significant difference in the parasitism rate between TD_h_ and TD_m_ (t = 1.67, df = 4, P > 0.05). The offspring female ratio of TD_h_ was significantly higher than that of TD_m_ (t = 8.96, df = 4, P < 0.01).

### The fecundity of *T. drosophilae*

**Figure 2:**
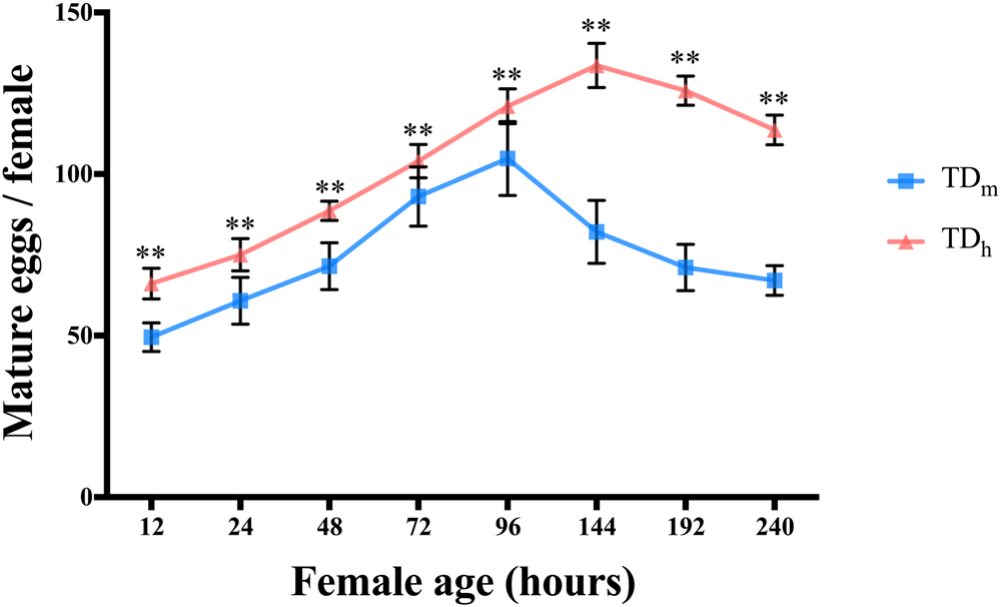
Egg maturation dynamics of female *T. drosophilae* when reared in two different hosts. Values are the means ± SEM. Significant differences at P < 0.05 are indicated by asterisks.

The number of mature eggs in the ovaries of TD_h_ and TD_m_ females was compared among different ages (Fig. 2). The results showed that the number of mature eggs was affected by the female age for TD_h_ (X^2^ = 69.06, df = 7, P < 0.01) and TD_m_ (X^2^ = 51.84, df = 7, P < 0.01). The mean number was further compared among different age classes using ANOVA between two *T. drosophilae* groups. Interestingly, the number of mature eggs of female TD_h_ and TD_m_ increased until the TD_m_ females were 96 hours old, whereas this increase persisted for an additional 48 hours for TD_h_. Thus, the maximum number of mature eggs of TD_h_ (133.60 ± 6.87) was observed 144 h after emergence, while that of TD_m_ (104.80 ± 11.44) was observed 96 h after emergence (t = 4.279, df = 7, P < 0.01; Fig. 2).

**Figure 3:**
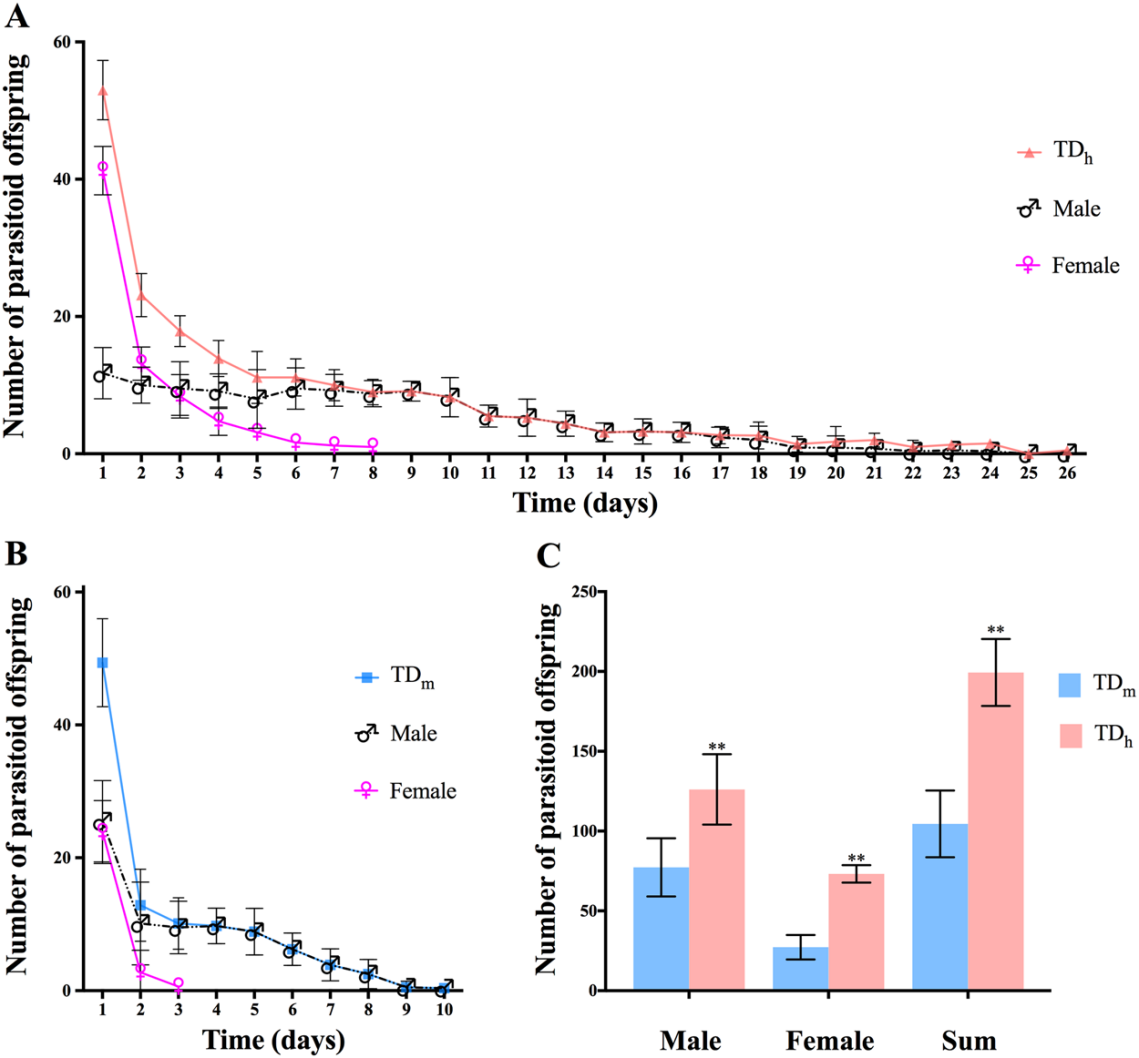
The offspring number of single TD_h_ and TD_m_ female wasps. (**A**) The number of offspring each day (red) was calculated by adding the male (black) and female (purple) offspring numbers. (**B**) The number of offspring each day (blue) was calculated by adding the male (black) and female (purple) offspring numbers. (**C**) The total number of offspring for a single TD_h_ and TD_m_ female. In total, 8 TD_h_ and 8 TD_m_ female wasps were used in this experiment, respectively. Values are the means ± SEM. Significant differences using Student’s t-test at P < 0.05 are indicated by asterisks.

We observed that mated TD_h_ continuously produced female offspring until 6 days after mating, compared with approximately 3 days for TD_m_ (Fig. 3A,B). In this assay, TD_m_ female wasps could survive 10.40 ± 0.91 days (Fig. 3B), whereas TD_h_ female wasps survived 23.81 ± 2.40 days (Fig. 3A). The lifetime number of emerged progeny was higher for the TD_h_ population (199.38 ± 20.94 offspring per female, n = 8) than for the TD_m_ population (104.50 ± 20.91 offspring per female, n = 8) (t = 9.07, df = 14, P < 0.01). The number of female progeny was 73.25 ± 5.47 for TD_h_ and 27.25 ± 7.69 for TD_m_ (t = 13.79, df = 14, P < 0.01) (Fig. 3C).

### The stress resistance ability of *T. drosophilae*

**Figure 4:**
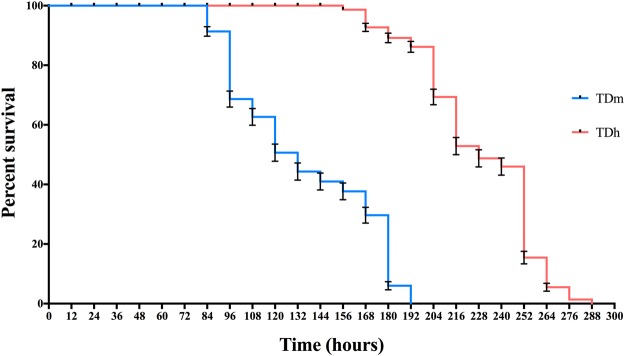
The survival rate of TD_h_ and TD_m_ during the starvation treatment. TD_h_ had a longer survival lifespan than TD_m_ under the starvation treatment (X^2^ = 744.30, df = 1, P < 0.01). Significant differences based on log-rank test (Mantel-Cox) analysis.

To determine *T. drosophilae* stress resistance ability, TD_h_ and TD_m_ were treated with different environmental stresses, including starvation and high and low temperatures. Under food deprivation, the starved TD_m_ wasps had a maximum life span of 192 hours, and half of the wasps survived 120 hours, whereas the TD_h_ wasps had a maximum life span of 288 hours, and half of the wasps could survive at least 216 hours. The TD_h_ wasps had a longer lifespan than the TD_m_ wasps under starvation treatment (Log-rank test X^2^ = 744.30, df = 1, P < 0.01) (Fig. 4).

**Figure 5:**
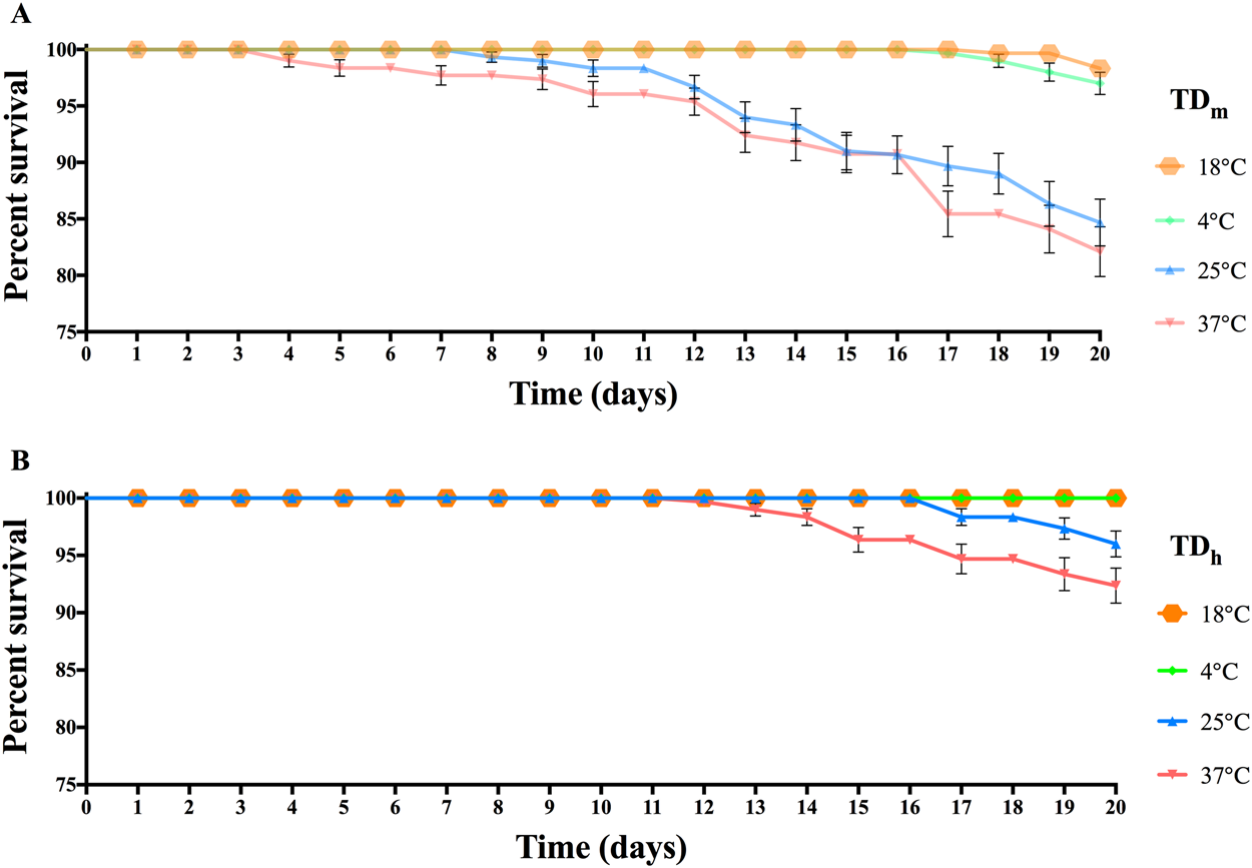
The survival rates of TD_m_ (**A**) and TD_h_ (**B**) at 4 °C, 18 °C, 25 °C and 37 °C. The results showed that almost all TD_h_ and TD_m_ wasps survived well at 4 °C and 18 °C. However, the survival rates of TD_h_ were higher than those of TD_m_ at 25 °C or 37 °C (25 °C: X2 = 23.09, df = 1, P < 0.01; 37 °C: X^2^ = 14.79, df = 1, P < 0.01). Significant differences were based on log-rank test (Mantel-Cox) analysis.

To determine how different temperatures affect *T. drosophilae* survival, we placed TD_h_ and TD_m_ into incubators at 4 °C, 18 °C, 25 °C and 37 °C. The results showed that almost all TD_h_ and TD_m_ wasps survived well at lower temperatures (4 °C and 18 °C). However, the survival rates of TD_h_ were higher than those of TD_m_ at 25 °C or 37 °C (25 °C: Log-rank test X^2^ = 23.09, df = 1, P < 0.01; 37 °C: Log-rank test X^2^ = 14.79, df = 1, P < 0.01) (Fig. 5A,B).

### *T. drosophilae* parasitism efficiency related to age

**Figure 6:**
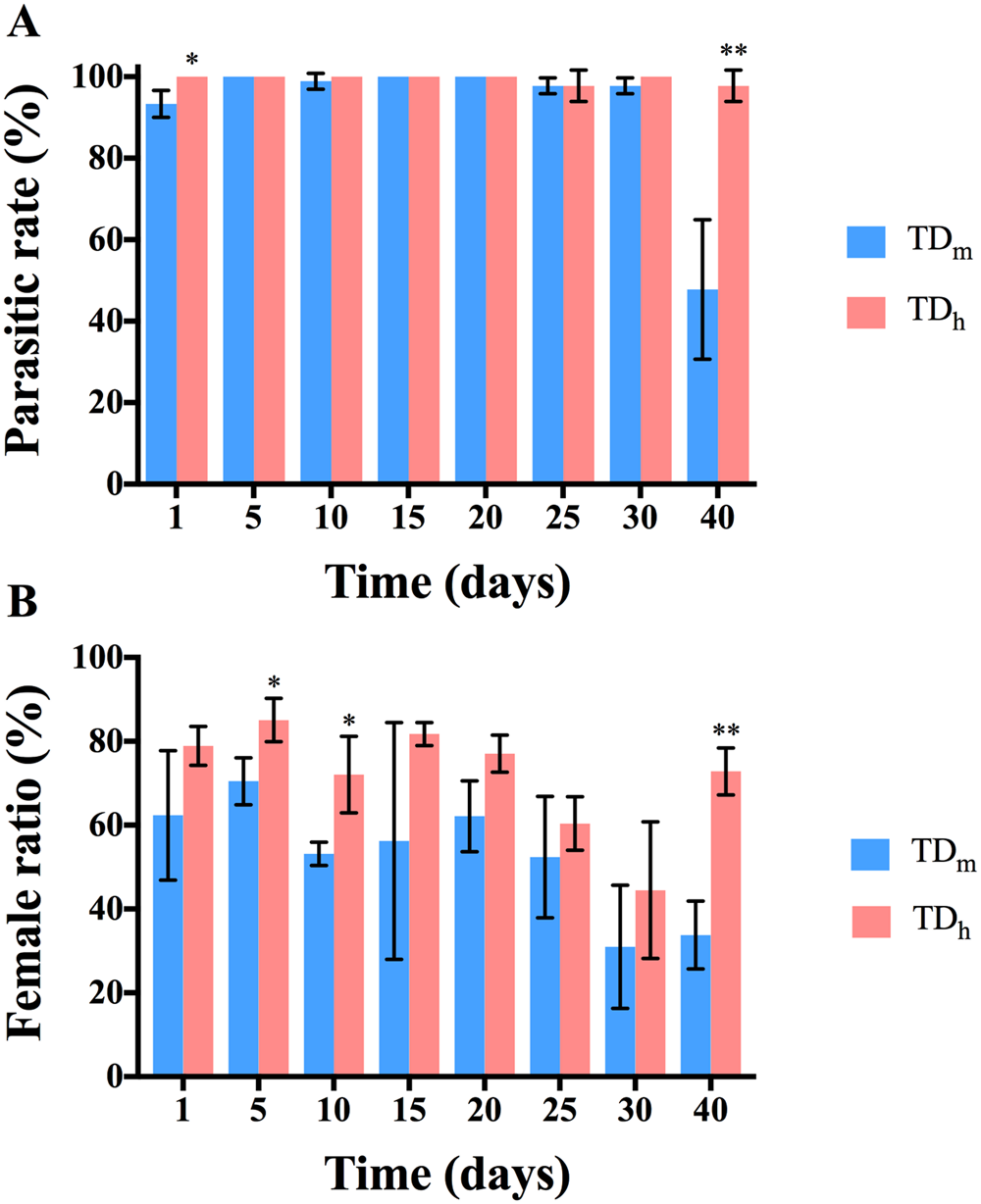
(**A**) The parasitic rate of TD_h_ and TD_m_ at different ages. There was a significant increase in the parasitism rate for the 40-day-old TD_h_ parasitoids compared to TD_m_ (t = 4.94, df = 4, P < 0.01) (**B**) The offspring female ratio of TD_h_ and TD_m_ at different ages. The offspring female ratio of TD_h_ was slightly higher than that of TD_m_; however, significant differences were found between TD_h_ and TD_m_ at 5 days (t = 3.32, df = 4, P < 0.05), 10 days (t = 3.43, df = 4, P < 0.05) and 40 days (t = 6.87, df = 4, P < 0.01) after eclosion. Values are the means ± SEM. Significant differences based on Student’s t-test at P < 0.05 are indicated by asterisks.

In order to evaluate the influence of *T. drosophilae* age on the parasitism rate, 1-, 5-, 10-, 15-, 20-, 25-, 30- and 40-day-old wasps were used to parasitize the hosts. The results showed that both TD_h_ and TD_m_ had an extremely high parasitism rate at all time points; however, a significant decrease in the parasitism rate was observed for the 40-day-old TD_m_ parasitoids compared with the 40-day-old TD_h_ parasitoids (t = 4.94, df = 4, P < 0.01) (Fig. 6A). In accordance with the results of our fecundity experiment (Table 1, Fig. 3C), the offspring female ratio of TD_h_ was slightly higher than that of TD_m_; significant differences were found between TD_h_ and TD_m_ at 5 days (t = 3.32, df = 4, P < 0.05), 10 days (t = 3.43, df = 4, P < 0.05) and 40 days (t = 6.87, df = 4, P < 0.01) after eclosion (Fig. 6B).

## Discussion

Assessing the capacity of the *T. drosophilae* parasitoid to attack Drosophilidae species and enhancing its ability to adapt to extreme environments are two of the most important steps for the release of *T. drosophilae* as a biological control agent. In this study, we showed that local *T. drosophilae* was able to successfully attack *D. melanogaster* and *D. hydei* under laboratory conditions. A previous study reported that *T. drosophilae* offspring reared in large hosts such as *D suzukii* were larger than those reared in *D. melanogaster*^14^. Because *D. hydei* had a larger size than *D. melanogaster*, we compared the offspring size that emerged from the two different hosts, and found that the size of TD_h_ was much larger than that of TD_m_.

Parasitoids reared in substitute hosts would help to increase the availability of biocontrol agents^20–22^. It has also been proven that large parasitoids of the same species have longer life spans, and large females produce approximately twice as many eggs as small females^18^. Thus, we evaluated the different parasitic characteristics of *T. drosophilae* reared in *D. hydei* and *D. melanogaster* pupae. Compared to *T. drosophilae* populations from California^14^, TD_m_ females in our experiments had a similar number of mature eggs, and the egg load increased during the first four days. However, the number of TD_h_ mature eggs was significantly higher than that of TD_m_ and increased during the first six days. Fecundity is the maximum potential reproductive output of a parasitoid female over its lifetime and represents one of the major parasitic characteristics. Under the test conditions, the daily fecundity of TD_m_ and TD_h_ decreased with increasing female age, and when provided only with *D. melanogaster* pupae, the adult female TD_m_ only survived for 10 days, which is shorter than the reported *T. drosophilae* lifespan^13^. However, TD_h_ survived for 26 days and produced more female offspring than TD_m_. Another interesting phenomenon was that female TD_h_ produced female offspring for 6 days after one mating event, compared with only 3 days for TD_m._ *T. drosophilae* has a sex-determination system in which males develop from unfertilized eggs and are haploid, whereas females develop from fertilized eggs and are diploid^23,24^. The results suggested that size differences of *T. drosophilae* between males or females from different hosts may influence sperm production or storage. In mosquitos, male size does correlate with total numbers of sperm within a male and the number transferred to females^25,26^.

Stress resistance ability is an important factor in evaluating parasitoid fitness and biocontrol efficacy in the field. A larger sized host may provide more nutrients that are vital for parasitoid development, which may be the reason why TD_h_ survived longer than TD_m_ in the starvation experiments. Additionally, our data indicated that both TD_h_ and TD_m_ wasps survived for a long time at lower temperatures (4 °C and 18 °C). The reason for this is that the lower temperature will slow the metabolism of the wasps and can even extend their lifespan^27^.

During the last 10 years, *D. suzukii*, also known spotted wing drosophila, has become widely distributed from Asia to Europe and North and South America^28–31^. *D. suzukii* has spread rapidly to become a serious pest that economically damages soft and thin-skinned fruits in the major fruit production areas^32–34^. Extensive applications of chemical insecticides will lead to a number of problems, such as pest resistance and chemical residue. Therefore, non-toxic and environmentally friendly biological control methods are urgently needed. Some entomopathogenic nematodes and fungi have been used to kill *D. suzukii* adults^30,35^. However, control of *D. suzukii* populations is very limited. So far, 50 hymenopteran parasitoids are reported to infect various drosophila species which belong to four families including two larval parasitoids, Braconidae and Eucoilidae, and two pupal parasitoids, Pteromalidae and Diapriidae^9^. Some studies have shown that most of these larval parasitoids cannot develop in D. suzukii because of its strong immune response^12^. *T. drosophilae* is a highly effective pupal parasitoid that can attack *D. suzukii* and has been proven to be a potential agent for biological control^14,36,37^. Our study demonstrates that *D. hydei*-reared parasitoids show more beneficial parasitic characteristics than *D. melanogaster*-reared parasitoids. *D. hydei* has a worldwide distribution and is easy to raise in large numbers. Therefore, rearing of *T. drosophilae* in *D. hydei* pupae could be a successful biocontrol strategy, especially for the aim of reducing *D. suzukii* infestation.

## Methods

### Insect collection and rearing

*D. melanogaster*, *D. hydei* and *T. drosophilae* were collected from traps baited with grape fruits in May 2016 at Zijingang Campus (30.29°N, 120.08°E), Zhejiang University, Hangzhou, China, and were maintained in our laboratory at a temperature of 25 ± 1 °C, relative humidity of 50–60%, and a photoperiod of 16 h: 8 h (L: D) inside plastic bottles (approximately 10 cm in length and 5 cm in diameter). Both *D. melanogaster* and *D. hydei* were maintained on a standard cornmeal/molasses/agar medium^38^. *T. drosophilae* colonies were maintained on *D. melanogaster* pupae, and the adult wasps were provided with apple juice/agar medium (27 g agar, 33 g brown sugar and 330 ml pure apple juice in 1000 ml diluted water).

### The parasitoid and host size measurements

*D. melanogaster* and *D. hydei* pupae, as the different hosts, were parasitized by *T. drosophilae*. For convenience, *T. drosophilae* that emerged from *D. hydei* and *D. melanogaster* pupae were called TD_h_ and TD_m_, respectively. The TD_h_ and TD_m_ adults and the pupae of their hosts were imaged using a KEYENCE VHX-2000C digital microscope system (Osaka, Japan). The body length and width of 18 *D. hydei* pupae and 37 *D. melanogaster* pupae were measured using KEYENCE VHX-2000C software. The length of the hind tibia or the length of the whole body is usually used as a proxy for the size of parasitoid wasps^14,39^. Here, body lengths of 10 female and 16 male TD_h_ and 12 female and 10 male TD_m_ were measured.

### Parasitism rate and offspring female ratio comparison

To compare the parasitism rate and offspring female ratio of TD_h_ and TD_m_, *D*. *melanogaster* pupae were parasitized by 4-day-old TD_h_ and TD_m_ similar to a previous study^14^ at a wasp/host ratio of 1:10 for 24 hours. This experiment was performed three times, and 200, 120 and 120 *D*. *melanogaster* host pupae were exposed to TD_h_ and TD_m_. The same approach was applied to compare TD_h_ and TD_m_ at different ages. After eclosion, TD_h_ and TD_m_ adult females were maintained on apple juice wasp food at 25 °C in an incubator without hosts. Then, 1-, 5-, 10-, 15-, 20-, 25-, 30- and 40-day-old TD_h_ and TD_m_ female wasps were collected to parasitize *D. melanogaster* pupae after fully mating with young TD_h_ and TD_m_ males, respectively, for 24 hours. Three replicates were performed for the experiments, and 5 females and 30 host pupae were used in each experiment. After being infected, the host pupae were kept in a 25 °C incubator until the wasps emerged. The parasitism rate and offspring female ratio of the wasps were calculated using the following formulas: parasitism rate = (the number of hosts − the number of emerged flies)/the number of hosts; offspring female ratio = the number of female parasitoids/the number of total emerged parasitoids.

### The fecundity and stress resistance ability of *T. drosophilae*

#### The egg load of a female parasitoid wasp

The newly emerged male and female wasps were collected and placed in plastic bottles containing apple juice wasp food without hosts. To compare the maximum egg load between TD_h_ and TD_m_, ovaries of 12-, 24-, 48-, 72-, 96-, 144-, 192- and 240-h-old female *T. drosophilae* adults were dissected in 1 × PBS buffer, pH 7.4. Ten female wasps for each category were dissected, and the mature eggs were counted at each time point. An egg was considered mature based on criteria used in a previous study^14^: the chorion of a mature egg is smooth, thin and transparent, and the developing embryo is visible, while immature eggs lack these characteristics and are attached to each another.

#### The offspring of a single female wasp

To compare the offspring numbers of TD_h_ and TD_m_, a fully mated female was allowed to parasitize 150 two-day old *D. melanogaster* pupae for 24 hours at 25 °C. Then, the host pupae were replaced by a new batch of 150 pupae the following day until the female adult died. The total number of offspring from single females was counted as the number of emerged wasps, including males and females. In total, 8 TD_h_ and 8 TD_m_ female wasps were used in this experiment, respectively.

#### Starvation and high and low temperature tolerances

One hundred newly emerged wasps of TD_h_ and TD_m_ (50 females, 50 males) were reared in an empty plastic bottle without any food at 18 °C for the starvation treatment. For the high and low temperature tolerance experiment, 100 newly emerged wasps of TD_h_ and TD_m_ (50 females, 50 males) were reared on apple juice wasp food in incubators at 4 °C, 18 °C, 25 °C and 37 °C. The survival rate (the number of surviving wasps/100) was calculated every 12 hours for the starvation treatment and daily for the high and low temperature tolerance analysis. Three replicates were performed for each experiment.

### Data analysis and statistics

The effects of female age on the number of mature eggs were analysed using a generalized linear model (GLM) and the mean number of mature eggs in different age classes were further compared using analysis of variance (ANOVA). Log-rank tests (Mantel-Cox) were performed to analyse trends in the survival rate during the environmental stresses, i.e., starvation and high and low temperatures. Student’s t-test was used to compare the body length or body width of parasitoids and hosts, the parasitism rate and offspring female ratio, as well as the fecundity of female parasitoid wasps. Statistical analyses were performed using GraphPad Prism version 7.0a (Graphpad Software, San Diego, CA) and SPSS software 25.0 (SPSS Inc., Chicago, IL). Error bars indicate the standard error of the mean (SEM), and all data sets are expressed as the mean ± SEM. Significant differences between groups were determined by the P-value and are marked with one asterisk for P < 0.05 and two asterisks for P < 0.01.
